# Osteopontin: Relation between Adipose Tissue and Bone Homeostasis

**DOI:** 10.1155/2017/4045238

**Published:** 2017-01-17

**Authors:** Carolina De Fusco, Antonietta Messina, Vincenzo Monda, Emanuela Viggiano, Fiorenzo Moscatelli, Anna Valenzano, Teresa Esposito, Chieffi Sergio, Giuseppe Cibelli, Marcellino Monda, Giovanni Messina

**Affiliations:** ^1^Department of Experimental Medicine, Section of Human Physiology and Unit of Dietetic and Sport Medicine, Second University of Naples, 16 Costantinopoli Str., 80138 Naples, Italy; ^2^Department of Medicine, University of Padua, Padua, Italy; ^3^Department of Clinical and Experimental Medicine, University of Foggia, Foggia, Italy

## Abstract

Osteopontin (OPN) is a multifunctional protein mainly associated with bone metabolism and remodeling. Besides its physiological functions, OPN is implicated in the pathogenesis of a variety of disease states, such as obesity and osteoporosis. Importantly, during the last decades obesity and osteoporosis have become among the main threats to health worldwide. Because OPN is a protein principally expressed in cells with multifaceted effects on bone morphogenesis and remodeling and because it seems to be one of the most overexpressed genes in the adipose tissue of the obese contributing to osteoporosis, this mini review will highlight recent insights about relation between adipose tissue and bone homeostasis.

## 1. Introduction

Osteopontin (OPN), also defined as secreted phosphoprotein-1 [1] (SPP1), sialoprotein-1 [1, 2], or early T lymphocyte activation 1 (Eta-1) [3] belongs to the small integrin-binding ligand N-linked glycoprotein (SIBLING) family [4]. SIBLING family consists of noncollagenous proteins (NCPs), primarily involved in bone metabolism and mainly located within the mineralized tissue such as bone and dentin, which also includes bone sialoprotein (BSP (IBSP)), dentin matrix protein 1 (DMP1), dentin sialophosphoprotein (DSPP), and matrix extracellular phosphoglycoprotein (MEPE) [5, 6]. These soluble secreted glycoproteins undergo posttranslational modifications. They are extensively and heterogeneously spliced, translated, phosphorylated, glycosylated, and proteolysed. These modifications give them bioactive properties [7]. SIBLING proteins exert biological roles in both paracrine and autocrine manner and through multiple functional domains may bind to cell surface integrins [7]. Originally described by Senger in 1979 as a secreted, 60-kDa transformation-specific phosphoprotein [8], human OPN is the most studied component of SIBLING family. It is a Arg-Gly-Asp (RGD) containing multifunctional soluble extracellular matrix-associated glycoprotein of 34 kDa [8] (apparent MW up to 75 kDa) [5, 6]. OPN has a gly-arg-gly-asp-ser (GRGDS) [9] cell binding domain with ~314 amino acid residues that can regulate cell activities through integrin receptors.

It is encoded by the* SPP1* gene, which maps to the long arm of chromosome 4 [6] as a tandem array and generates three splice variants of mRNA, including the isoforms OPN-A, OPN-B, and OPN-C. OPN-A constitutes the full-length variant [2], whereas isoforms B (missing exon 5) and C (missing exon 4) are the splice variants [1, 2]. These different forms can display specific expression and have distinct biological roles in different cell contexts. The name “osteopontin” was chosen by Heinegard's group that cloned the protein from the rat osteosarcoma. OPN is produced by cells located in the osteoid matrix, and its name “osteopontin” denotes the property of acting as a bridge (“pons” in Latin) between cells and hydroxyapatite of bone [2, 6]. OPN exists as a secreted (sOPN) and intracellular form (iOPN) [2], being translated from different start codon in the single OPN mRNA.

Intracellular OPN was initially described by Sodek's group in rat calvarial cells [2, 3].

Although OPN exists intracellularly as a regulator of cytoskeleton dynamics and gene expression, most studies have focused on the secreted form.

## 2. Osteopontin Functions

OPN is a multifunctional protein widely distributed in many tissues and body fluids, such as plasma, urine, milk, and bile [10, 11]. It is produced by several cell types, including immune cells like activated macrophages and T cells, cells of brain and kidney, vascular smooth muscle cells, bone marrow myoblasts, dendritic cells, hepatocytes, and neural cells [2, 6, 9]. Furthermore, OPN is produced by cells involved in bone morphogenesis such as osteoblasts and osteoclasts. OPN regulates various biological activities including matrix remodeling and tissue calcification, monocytes/macrophages migration and chemiotaxis, production of a variety of proinflammatory cytokines and chemokines, and inhibition of apoptosis activities [6]. Furthermore, it is involved in pathophysiological processes as malignancy, insulin resistance and type 2 diabetes, autoimmune disorders, atherosclerosis, steatotic hepatitis, end-stage kidney failure, response to stress, obesity-induced inflammation, and osteoporosis [3, 8]. Recently, numerous researches have examined whether there is a relationship between obesity and osteoporosis. Obesity and osteoporosis are two related polygenic disorders of body composition and represent a major health threat worldwide, with high impact on both morbidity and mortality [12, 13]. Interestingly, an increase of OPN levels in serum and cerebrospinal fluid was found in Alzheimer's disease (AD) patients [14]. AD is a chronic neurodegenerative disease and is the cause of 60% to 70% of cases of dementia [15–18]. Conversely, a decrease of OPN levels was associated with the improvement of cognitive functions [19, 20].

At bone level, OPN has multifaceted effects on morphogenesis and remodeling [3, 6, 13, 14], being associated with bone turnover and bone mineral density (BMD). Experimental evidence suggests that the gene of OPN is one of the most overexpressed genes in the adipose tissue of obese patients [1, 8].

## 3. Fat-Bone Relation

The relationship between obesity and skeleton is complex and not yet fully understood. Body mass index (BMI) is generally recognized to have a good positive correlation with bone mineral density (BMD) as measured by central Dual-Energy X-Ray Absorptiometry (DXA) [21]. For a long time it has been thought that obesity was apparently beneficial for bone metabolism [22, 23], particularly in women after menopause, playing a protective effect against the development of bone loss and osteoporosis. Adiposity might ensure bone health probably for the relative hyperestrogenism produced by adipocytes from androgen precursor in postmenopausal women [24–26]. Also the well-established positive effect of mechanical loading (muscle contraction and gravitational loading) related to body weight might positively influence BMD, by decreasing apoptosis and facilitating osteogenic differentiation through the activation of Wnt/b-catenin signaling pathway [27–31]. However, in last decades the view that obesity has an protective effect against osteoporosis has been questioned [32, 33]. Some reports have shown that excessive visceral fat and fat mass have negative effects on bone health, being associated with low total bone mineral density and content [34–36]. According to these results, another study conducted in healthy premenopausal women demonstrated that central adiposity was associated with low bone quality [37]. Furthermore, it has been suggested that in men and pre- and postmenopausal women fat mass plays a key role in bone health, representing the significant determinant of BMD at the lumbar spine and proximal femur [19, 22]. Similarly, Greco et al. [38], in a cross-sectional study, evaluated 340 obese women and showed that trunk fat negatively correlated with BMD playing a detrimental role in skeletal metabolism in terms of low BMD, bone markers, and systemic factors influencing bone tissue.

Interestingly, Papakitsou et al. [39] found in obese women a lower rate of bone formation as measured by the serum concentration of type I collagen propeptide, suggesting that obesity can inhibit the production of new collagen. Finally, recent in vitro data supported the hypothesis of decreased osteoblast activity in subjects with increase of trunk mass because of alterations of the Wnt/*β*-catenin pathway [40]. Therefore, obesity and fat accumulation might be detrimental to bone health. However, further investigations are required to better understand the complex pathophysiological relationship between body weight and BMD. Different theories have been proposed to explain the complex interplay between adipose tissue and bone [16, 17]. It has been proposed that the pathophysiological relevance of adipose tissue in bone turnover resides in the participation of three possible mechanisms. (1) There is an intimate link between adipocytes and osteoblasts related to their common origin from a progenitor cell, the pluripotential mesenchymal stem cell (MSCs) that resides in a specialized bone niche [41–43]. As a common precursor, the controlled lineage commitment of MSCs plays an important role in the maintenance of skeleton homeostasis. Osteoblast/adipocyte differentiation, although not mutually exclusive, is intertwined and highly regulated by several cell-derived transcription factors [44] and has been implicated in modification of bone remodeling. For example, recently Yeung et al. [45] have demonstrated that postmenopausal women have more than twice bone marrow fat compared with premenopausal women. (2) Adipose tissue is not only a passive energy reservoir but also an active, complex endocrine organ [16, 17] that plays an important role in regulating whole body homeostasis. Adipose tissue secretes various signaling molecules and bioactive compounds, named adipokines (leptin, resistin, IL6, osteopontin, etc.). Adipokines influence bone remodeling (obesity of bone) through upregulated proinflammatory cytokine production and exert an indirect effect on the sympathetic tone via hypothalamic nuclei. Expression array studies on human adipose tissue have pointed out an activation of several inflammatory pathways in obesity [46]. In fact, chronic low-grade inflammation is associated with adipose tissue expansion in obesity and it is determined by increased systemic concentrations of proinflammatory endocrine cytokines, such as TNF-alfa [47], and osteopontin [1, 33] in patients and animal models of obesity. These cytokines may uncouple bone remodeling by stimulating osteoclast activity or inhibiting bone formation modifying the receptor activator [16]. Thus, all these mechanisms may represent a crosstalk between adipose tissue and skeleton, detrimental for bone health. The exact nature of factor that triggers this mechanisms is unknown.

Osteocalcin is a 49-amino acid bone matrix noncollagen protein produced by osteoblasts, which is involved in bone deposition and calcium homeostasis. Studies show that osteocalcin also has an important role in energy homeostasis and glucose metabolism: in cross-sectional and prospective epidemiological studies, circulating osteocalcin levels are inversely associated with the risk of type 2 diabetes [48, 49], metabolic syndrome [50, 51], overall/abdominal adiposity and insulin resistance [52], and reduced BMD [38].

Furthermore, OC levels were positively associated with cognitive performance in older nondemented women [53–56]. Genetic osteocalcin deletion induced glucose intolerance, increased fat mass, insulin resistance, decreased expression of insulin target genes in liver and muscle, and decreased adiponectin gene expression in adipose tissue. In contrast, recombinant osteocalcin administration improved insulin secretion and sensitivity and prevented high-fat-induced obesity and diabetes.

In addition to improving pancreatic insulin secretion, experimental models show that osteocalcin can protect against high-fat-induced obesity, insulin resistance, and nonalcoholic fatty liver disease (NAFLD) [57, 58]. Epidemiological studies are sometimes conflicting and require further validation [59]. Currently the osteocalcin is emerging as an important modulator of energy homeostasis and the metabolism of glucose in various tissues, raising the possibility that this bone-derived hormone may become a new treatment for obesity-related disorders, a hypothesis currently being tested.

## 4. Dysregulation of Osteopontin

In mouse models and obese humans, OPN is one of many inflammatory molecules overexpressed in adipose tissue [60, 61]. Besides, its levels showed a great difference in adipose tissue between monozygotic twins discordant for obesity condition [62]. Its overexpression in obese adipose tissue is mainly attributable to accumulation of the macrophages [1] and other inflammatory cells, stromal vascular cells, and adipocytes. Nomiyama et al. [63] suggested that OPN might be involved in linking obesity-induced inflammatory processes and metabolic changes in adipose tissue. Similarly, it has described a high level of OPN RNA expression in adipose tissue of obese insulin resistant rats and humans [8]. Kiefer and colleagues [1] showed that obesity is associated with a striking increase of OPN expression selectively within adipose tissue. Adipose tissues are the major source of OPN and also of its obesity-induced upregulation. These data point toward a specific pathophysiological role of OPN in obesity. Therefore, OPN could be a key regulator of inflammatory processes linked to obesity-induced adipose tissue inflammation and become a major target for treatment of adipose tissue inflammation-related disorders. Recently, it has been shown that genetic OPN deficiency and antibody-mediated neutralisation in mice improve inflammation and protect from obesity and insulin resistance induced by a high-fat diet [46]. OPN would act as proinflammatory cytokine and have pleiotropic function in inflammation. The primary role of OPN seems to facilitate recovery of the organism after injury or infection [38]. However, several studies have highlighted the critical role of OPN in regulating not only adipose tissue inflammation, but also insulin resistance and diabetes mellitus [64, 65].

Recently, an alteration of adipose tissue function has been related to various human metabolism disorders such as osteoporosis [66]. It has been suggested that OPN influences adipogenic process, in bone marrow of obese women, contributing to the development of osteoporosis. An imbalance has been described between normal adipogenesis and osteogenesis of MSCs, prevailing adipocyte differentiation on osteoblast differentiation [67]. MSCs isolated from bone marrow in postmenopausal patients with osteoporosis express more adipocytic differentiation markers than those in women with normal bone mass [68]. Meunier et al. [69] analyzed 81 iliac crest biopsies from elderly women and observed a considerable amount of fat in bone marrow of women with osteoporosis, relative to levels of fat in healthy young women. This observation was confirmed in subsequent studies [70–72]. An increment of marrow adipocytes was observed also by Schellinger et al. [73]. After oophorectomy, rats showed an increase in the amount of fat in the bone marrow [17]. Fat infiltration might considerate a typical example of lipotoxicity. Triggiani and colleagues [30], too, have observed similar results.

Many researchers considered osteoporosis as a faultiness of bone marrow MSCs [74] in differentiating into other cell lineages, with adipogenesis being enhanced compared to osteoblastogenesis. Molecular mechanisms of adipocyte differentiation have been studied using both in vitro and in vivo models but the cellular and molecular mechanisms mediating the pathological switch in fat remain to be determined [39]. What is the player of the fat-bone axis in the bone marrow? Because OPN−/− mice could be due to changes in the balance between osteogenic and adipogenic differentiation by MSCs [3], we want to know the role of OPN in MSCs differentiation. Further studies are needed to define the precise relationship between obesity and osteoporosis. Previous studies have shown that, in overweight and obese humans, the dysregulation of circulating signaling factors, such as inflammatory cytokines, might play an important role in changes of MSC differentiation [75–78]. Among cytokines, OPN may represent a multifaceted regulator between fat and bone on bone remodeling and contribute to the development of osteoporosis [3]. In the study conducted by Inoue and Shinohara [3] on the effects of secreted OPN in regulating MSC differentiation, the authors revealed an important role of OPN-integrin *αv*/*β*1 in regulating adipogenic and osteogenic differentiation. Di Bernardo et al. [78] in their study in vitro observed that the incubation of MSCs with the sera of overweight individuals promoted a bias in differentiating MSCs in adipogenic line, probably for a modification of circulating cytokines. OPN by itself induces expression of a variety of proinflammatory cytokines and chemokines being an important regulator for initiating adipose tissue macrophage and macrophage-like cells infiltration, triggering a vicious circle in which inflammatory cytokines play important roles in MSC differentiation. Moreover, other studies are required to elucidate the precise role of OPN in the pathophysiology of osteoporosis and as a possible crucial link of bone-adipose axis.

## 5. Conclusion

OPN was identified in 1980; it is a key regulator of many metabolic and inflammatory diseases, such as diabetes, cardiovascular disease, and obesity. Some studies have shown that OPN is causally involved in the pathogenesis of insulin resistance and type 2 diabetes, while other studies have shown that OPN is a protective islet protein preserving insulin secretion. In addition, experimental and epidemiological evidence emerging disclosed complex cytokine and hormonal crosstalk between bone cells, liver, and adipose tissue, which adjusts coordinated bone remodeling, energy metabolism, and glucose homeostasis; changes in this network may contribute to the pathogenesis of obesity and related disorders [58], but some key questions have not yet been resolved. Therefore, further research will clarify the clinical significance of these changes that cause metabolic and inflammatory diseases, and they will identify individuals at higher risk for developing such complications and for therapeutic purposes.
